# “Typical” chiropractic patients– can they be described in terms of recovery patterns?

**DOI:** 10.1186/s12998-017-0152-0

**Published:** 2017-08-09

**Authors:** Iben Axén, Charlotte Leboeuf-Yde

**Affiliations:** 1 Institute of Environmental Medicine, Unit of Intervention and Implementation Research for Worker Health, Karolinska Institutet, Nobels väg 13, 171 77 Stockholm, Sweden; 2 Institute for Regional Health Research, University of Southern Denmark, B.Winsløws Vej, 19, DK 5000 Odense C, Denmark

**Keywords:** Recovery, Course, Evidence-based, Chiropractic, Low-back pain, Spinal manipulation

## Abstract

**Background:**

Chiropractors expect the typical patient to recover fully or to improve quickly with treatment if relapses occur. However, a mismatch between expectations and outcome would have a negative effect on both the chiropractors’ professional self-esteem and patients’ satisfaction with care.

The prevalence of three types of recovery patterns among patients with non-specific low back pain (LBP) was calculated: 1: A full sustained recovery. 2: Initial recovery, but with one or several relapses followed by a period of recovery. 3: No initial recovery, but at least one period of recovery during the six month course of the study. Also, the number of patients classified as recovered at the end of the study was calculated.

**Method:**

In this Swedish clinical observational study from 2008 to 2009, an automated text message system (SMS-Track®) was used to ask chiropractic patients weekly for 6 months about the number of days their LBP had bothered them during the past week. Data were analyzed blindly by two researchers by viewing individual graphs and was performed twice.

**Results:**

In all 176 of 262 patients with non-specific LBP were included in the analysis. 1) Twenty percent of patients (CI: 15.3-26.9) made a full sustained recovery. 2) A further 20% (CI: 14.8-26.2) recovered initially but experienced a relapse, followed by at least one new period of full recovery. 3) Twenty-three percent (CI: 17.3-29.3) of patients failed to recovery initially but experienced recovery some time during the study. 4) Thirty-seven percent (CI: 30.3-44.1) had no periods of recovery, and were therefore classified as having a non-favorable course. At the end of the study, 41% (CI: 34.1-48.1) were classified as recovered.

**Conclusions:**

The results from this study can be used to introduce a realistic approach to chiropractic care in LBP, which should reduce disappointments among both chiropractors and patients. There was no “typical” recovery pattern. Trajectories were, in fact, spread over several subgroups with somewhat more than half reporting a favorable course but only one fifth enjoyed early and sustained recovery. Further, from a research perspective, the end-of -study status does not well depict the outcome experienced by patients.

## Background

Clinical decisions are partly based on symptoms, signs and findings, but also on clinicians’ knowledge and experience regarding clinical outcome. The information conveyed to patients concerning the likely development of the condition will affect patients’ satisfaction with treatment, and even clinicians’ satisfaction with their work. It seems reasonable to assume that the bigger the gap between expectations and outcome, the more dissatisfaction for patients and clinicians alike.

Low back pain (LBP) is commonly treated by chiropractors [1, 2]. Patients with LBP who seek chiropractic care may present with short-lasting as well as long-lasting problems [3]. Swedish chiropractors were found to have high expectations regarding improvement, more so than their patients [4]. Chiropractors seem to assume that if the treatment is ‘correct’ and the patient follows the treatment plan, it will work out well. This view is not surprising, as clinicians often release their patients when they feel better. The logical conclusion made by the clinician would be that when a patient is not seeking active care, he/she is doing well. In a Norwegian study, chiropractors were asked one year after patients’ initial consultations, if they thought the patient had been symptom free or not with an option to answer “don’t know”. The vast majority of patients (74%) were by the chiropractors described as having been symptom free, whereas the vast majority (80%) of the patients themselves reported having had relapses [5]. In other words, the chiropractor considers this to have been a success whereas the patient probably does not.

Clearly, for the clinician, information about the period after treatment is difficult to ascertain. Tracking patients after consulting for care with weekly recordings is, however, possible via automated text messaging and mobile phones. In a Danish study using this technology, SMS Track [6], the level and development of improvement was found to differ between different subgroups of patients over a period of 18 weeks [7].

The existence of different courses was also confirmed in a Swedish study using the same system to collect weekly data but over 6 months [8]. In the latter study, subgroups were identified on the basis of their pain development over time, but it was noted that the majority of patients would be considerably improved overall. In both studies [7, 8] it was shown that, for the majority of cases, recovery was not complete, as the weekly data rarely indicated complete absence of LBP.

Similar findings have been observed for LBP patients in primary care [9, 10]. However, detailed developments are not possible to study using conventional trial methodology, which measures status at baseline and then at follow-up some (usually) considerable time later.

In the above studies, results were presented on a group level. Although summary statistics are helpful to gain an overall understanding, clinicians deal with individuals and want to view data on an individual level. For this reason, we studied individual data collected weekly over 6 months on chiropractic patients with LBP from the Swedish study. These data made it possible to visualize the development of the number of days with “bothersome” LBP each week, for each patient.

The outcome ‘recovery’ has been suggested by de Vet et al. [11] to be a period of at least 4 weeks without pain. This definition has been tested in cohorts from the general population [12], from primary care [13] and from secondary care [14], and found to be useful. Thus, in the general population, recovery from LBP has been shown to be common [12], in primary care less common [13], and in secondary care, rather uncommon [14]. Therefore, this definition can now be considered to be evidence-based.

Using the clinical perspective, we were interested in various patterns of recovery, namely to identify: 1: Patients who recovered and then reported sustained recovery. 2: Patients who made initial recovery, then had a relapse but experienced recovery again. 3: Patients who did not report initial recovery but nevertheless fulfilled the criterion for being ‘recovered’ at some point during the course of the study. 4: Finally, patients who did not recover by this definition. Lastly, as most studies measure outcome at the end of the study only, for comparison, we also calculated the prevalence of patients who reported to be ‘recovered’ at the end of the study, regardless their pattern prior to that.

The overall aim of the study was to see if any of these recovery patterns was sufficiently frequent to describe the “typical” chiropractic patient.

## Method

### Study participants, data collection and ethical considerations

Data were available from a Swedish multi-center prospective study where 262 chiropractic patients with LBP were enrolled consecutively by 35 chiropractors. Details of the participating chiropractors, the study sample, collection of data and feasibility of the data collection method has been given elsewhere [15]. For a descriptive summary, please see Table 1.

**Table 1:** Description of the study sample at baseline, and of the respondents with high compliance (answering 21 weeks or more)

	All patients recruited *n* = 262	Respondents with high compliance *n* = 176
Gender (%) Male	52	52
Age, mean, (range)	44 (16-69)	45 (21-69)
Pain, mean, (SD)	4.40 (2.24)	4.41 (2.13)
Leg pain (%)	50	50
Pain duration (%) > 30 days	57	57
Self-rated health, EQ-5D, mean (range)	0.781 (0.715-0.836)	0.781 (0.722-0.836)

In short, patients should be of working ages and have non-specific LBP with or without leg pain. They should not have been treated by a chiropractor for the past three months, be able to communicate in Swedish, have access to a mobile phone and be knowledgeable about sending and receiving text-messages. The clinicians noted down baseline information (age, gender, pain intensity, presence of leg pain, duration of LBP the previous year, self-rated health) and a questionnaire was sent to the participants after six months (concerning self-rated health). The treatment content was decided in each case by the treating chiropractor and was not recorded for the purpose of this study. Data collection took place between May 2008 and June 2009.

An automated text message system (SMS-Track, (6)) was used to ask the participating patients about their LBP the previous week. This was done weekly for 6 months. The text message question was *“How many days during the previous week has your low back pain been bothersome, (i.e. affected your daily activities or routines)? Please answer with a number between 0 and 7*”. The patients responded by sending a reply text message with a number corresponding to the number of days they had experienced bothersome LBP. Answers went directly into a data file suitable for analysis.

The concept of bothersomeness has previously been studied by Dunn et al., who found that it correlates with pain, disability and psychological health [16]. In this study, it was used as a proxy for the impact of pain as questions asked in the text message system were confined by length.

The study was approved by the ethics committee at Karolinska Institutet, Stockholm, Sweden (2007/1458-31/4). All patients gave written informed consent to participate.

### Definitions of terms describing a favorable course, involving recovery

In the analyses, a week without pain was defined as reporting zero number of days with bothersome pain. ‘Recovery’ was defined as at least four weeks in a row without LBP following de Vet et al.’s consensus recommendations [11]. ‘Sustained recovery’ was defined as no further weeks with LBP following the initial recovery. A ‘relapse’ was defined as any reported number of bothersome pain (i.e. 1-7 in any week) following the initial period of recovery. Missing data were interpreted as weeks with LBP unless there was one single week of missing data in a row of many similar values, in which case it was interpreted as a week with that same value.

A sensitivity analysis was also performed using a more lenient definition of a week without LBP. A week with 2 days or less of bothersome LBP was arbitrarily used as a clinically sensible option, and all the analyses were performed a second time.

### Analysis of data

Patients for whom more than 20% of the weekly text-message replies were missing were excluded from the analyses to secure solid estimates. In previous analyses, compliance was found not to be associated with age, sex or season [15]. This selection of compliant respondents was therefore not believed to be a source of bias. For the remaining study sample, four subgroups were defined: 1) Patients with sustained recovery, 2) Patients who recovered initially, but had a relapse, followed by at least one period of recovery, 3) Subjects who did not have an obviously favorable course of recovery initially but who, nevertheless at some time during the study period, reported at least one period of recovery, and 4) Patients who did not recover, i.e. who never reported four weeks without pain.

These categories were chosen from a clinical perspective, i.e. according to what a clinician may observe when communicating with a patient. Analysis was made through visual inspection of all individual course patterns, as the task concerned a limited number of curves and was relatively quick. It was done twice by both authors, the second time blind to the initial findings to be able to identify and correct any errors.

For comparison, the prevalence of patients who reported recovery at the end of the study was also calculated.

Results were reported as percentages with their 95% confidence intervals for each group.

## Results

In all, 176 individual course patterns were included in the study as they had excellent compliance, having produced text-message responses in at least 80% of the 26 weeks of the study. The participants were aged between 21 and 69 (mean and median 45) and 52% were men. Fifty-seven percent had experienced LBP for altogether more than 30 days during the year preceding the first consultation.

Intra-examiner reliability of the scoring into course patterns was close to perfect, as there was disagreement in only two cases between the two times the analyses were performed (i.e. 174 out of 176 = 99% agreement).

### Patients with sustained recovery (20%)

**Fig. 1:**
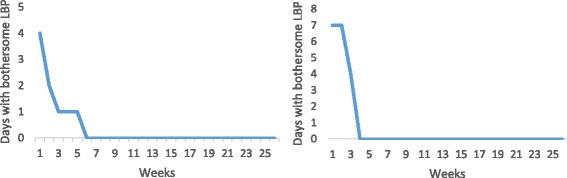
Examples of individual pain trajectories of patients with LBP who were recovered (reporting less than 0 days of bothersome pain per week for 4 consecutive weeks) quickly (in less than 2 months) after consulting for care and remained recovered (reporting no further weeks with bothersome pain throughout the study period)

**Fig. 2:**
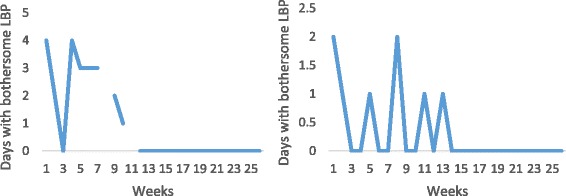
Examples of individual pain trajectories of patients with LBP who were recovered (reporting no days of bothersome pain per week for 4 consecutive weeks) slowly (more than 2 months after consulting for care) and remained recovered (reporting no further weeks with more than 1 day of bothersome pain throughout the study period). Where the line is broken, data are missing

Twenty percent (CI: 15.3-26.9) of the cohort were classified as recovered with sustained recovery for the rest of the study period. Forty-four percent of this group showed recovery before the end of the second month. Some examples of such patients are shown in Fig. 1. The remaining subjects in this group reported recovery at a slower rate. Some examples of these profiles are shown in Fig. 2.

### Patients with recovery, followed by a relapse followed by at least one period of recovery (20%)

**Fig. 3:**
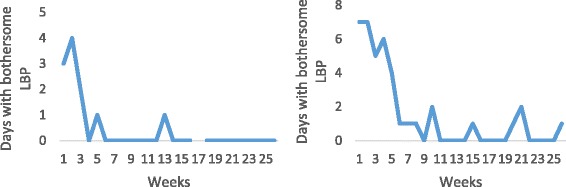
Examples of individual pain trajectories of patients with LBP who were recovered (reporting no days of bothersome pain per week for 4 consecutive weeks) after consulting for care but had at least one relapse (reporting more than 1 day of bothersome pain) followed by at least one period of recovery. Where the line is broken, data are missing

As in the first group, 20% (CI: 14.8-26.2) of the patients experienced initial recovery (either quickly or slower) but had at least one relapse followed by at least one period of recovery. For examples, see Fig. 3.

### Patients with at least one period of recovery at some time during the study period (23%)

**Fig. 4:**
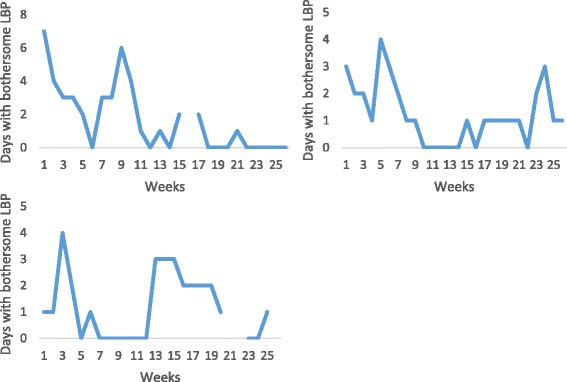
Examples of individual pain trajectories of patients with LBP who, at some time during the study, reported at least 4 weeks without LBP but without sustaining this level. Where the line is broken, data are missing

The third group consisted of 23% (CI: 17.3-29.3) of patients who, at some time during the study, reported at least 4 weeks without LBP but without sustaining this level. We noted several types of patients. For example, there were those, whose favorable course occurred late in the course (the first example in Fig. 4). Other examples are patients who had an initial favorable course that was interrupted by a relapse that occurred so late in the study that any new period of recovery would not have time to occur within the study (the second example in Fig.4). Finally, some patients had one or several pain-free intervals but in the “wrong” places (the third example in Fig. 4).

### Patients who did not recover (37%)

**Fig. 5:**
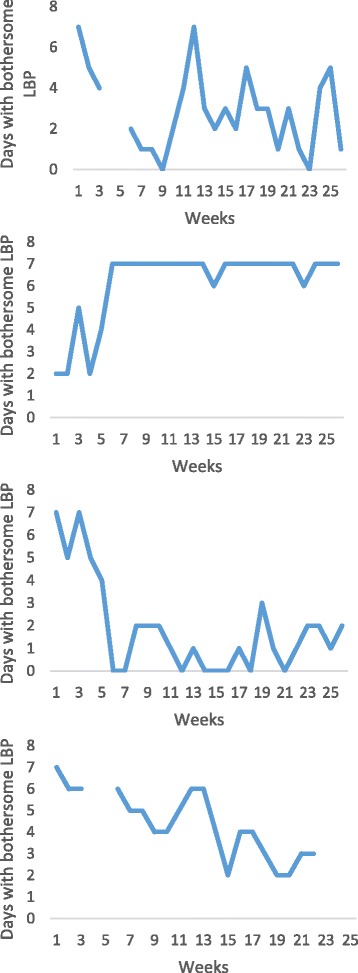
Examples of individual pain trajectories of patients with LBP who did not fit any of the above categories of recovery. Where the line is broken, data are missing

Thirty-seven percent (CI: 30.3-44.1) of the patients could not be classified into any of the above categories of recovery. These were patients i) who either did not fit into our definition of recovery (as illustrated in the first example in Fig. 5), ii) who seemed to get worse (second example in Fig. 5), iii) whose periods of improvement were too short-lasting to qualify as recovery (the third example in Fig. 5), or even iv) patients who appeared to get better but without reaching our definition of recovery (fourth example in Fig. 5).

### Patients classified as recovered at the very end of the study (41%)

At the time of the last (26th) week of the study, 41% (CI: 34.1-48.1), could be classified as ‘recovered’, i.e. reporting to have been free of LBP for at least 4 weeks.

In summary:

- 20% (CI: 15.3-26.9) of the cohort fulfilled the criteria for sustained recovery, whether quick or slow in the beginning.
- 20% (CI: 14.8-26.2) of the cohort were classified as recovered but experienced a relapse, followed by another period of recovery.
- 23% (CI: 17.3-29.3) of the cohort had at least one period of recovery but could not be fitted into any of the previous two groups.
- 37% (CI: 30.3-44.1) of the cohort did not belong to any of the above categories, meaning that most of these individuals did not have an obviously favorable course.
- 41% (CI: 34.1-48.1) of these individuals could be classified as recovered by the end of the study without taking into account the trajectory during the first five months.

A sensitivity analysis, using 2 pain days or less as the marker for recovery, showed that patients reporting sustained recovery more than doubled to 48% (CI: 41.2-55.5), a few more 26% (CI: 20.3-33.0) had a relapse after recovery, fewer patients 13% (CI: 9.4-19.4) experienced a single episode of recovery, and only 13% (CI: 9.0-18.7) were not recovered by this definition.

## Discussion

### Major findings

Two major lessons were learnt from this study. First, the “typical” recovery pattern of a chiropractic patient with LBP does not exist. Sixty-three percent of the patients will experience recovery, but recovery was sustained only in a third of these cases. The remaining 37% seem to be rather more complicated in that they do not have a clear pattern of quick, steady or prolonged improvement, and could be described as having persistent pain.

The second lesson is that the status of ‘recovered’ at the end of the study [11] did not correspond well with the course as viewed over the entire study period. Although 41% would have been classified as recovered with a classical study design (one assessment at the end of the study period), the more detailed analyses told a different story. This corresponds to previous findings that outcomes in fluctuating conditions need to be monitored repeatedly [17].

### Comparison with other studies

We know of one similar study where trajectories based on weekly pain reports were analyzed visually in a group of chiropractic patients [7]. However, this was a shorter study (18 weeks) and a different definition was used for recovery. Still, improvement was found in the same proportion (63%) of patients.

Different patient populations may or may not have similar findings. For example, in a cohort in ordinary primary care, 30% of patients with LBP were classified as recovered, and only 13% as fluctuating [16] over one year. The rest of the individuals were classified as persistent (mild or severe).

### Methodological considerations

The major strength of this study was that it used data collected with weekly text-messaging to establish the clinical course in patients with LBP. This limits the amount of memory decay that can take place over time and the individual course can be studied for each patient in detail. The advantage of using mobile phones rather than, for example, computer-based diaries, is that most people have a mobile phone. As such, the data collection tool is always available, which was reflected in the high compliance. However, everybody did not answer their text message every week. Our 176 participants were selected because they had been compliant at least 80% of the time. This was necessary in order to secure solid estimates of the development. Therefore, we do not know the recovery profile for the poor compliers of this study. Some were probably not improved at all whereas others may have been well and therefore not keen to continue with the text-messaging. A previous analysis of the drop-outs in this cohort indicated that leaving the study was unaffected by age and sex and that the clinical course of poor compliers resembles that of the high compliers [18]. No record was kept of eligible patients refusing participation, and therefore, the possible selection bias cannot be studied. Nevertheless, all in all, we believe that our data provided a fair representation of the clinical course of patients with LBP who consult chiropractors.

Another strength, was that the study participants were treated by chiropractors in a multi-center practice-based study, which ensured a wide variety of patients and treatment that was fairly typical of what happens in real life.

Text message questions must be brief, easy to answer and few. This makes it difficult to study multiple aspects of a disease. In our study, patients were asked to report pain that bothered them. The concept of bothersomeness has been found to summarize symptoms [19] and by adding a definition of activity limitation [20], it was thought also to be a proxy measure for pain severity. Therefore, this question was intended to provide a useful answer that would not register minor moments of pain but LBP of a certain magnitude and with some obvious consequences to the patient.

We based our calculation of presence/absence of LBP on the number of days per week with bothersome LBP. However, it would be possible for patients to experience improvement in the level of pain intensity or of the threshold of pain appearance without experiencing a diminished number of days with bothersome pain. Such information could not be captured with our outcome measure. However, according to a previous comparison between the level of pain and number of days with pain, performed in a chiropractic patient population with LBP, the course profiles for these two variables were found to be almost identical [21], indicating that pain intensity and days with bothersome pain follow the same pattern, at least in this type of patient population.

We used the consensus definitions for recovery and new episode proposed by de Vet et al. [11] in a somewhat modified form, as we consider these evidence-based due to the results obtained in the previous studies from primary [13] and secondary [14] care as well as from the general population [12]. Other definitions of any or all of the concepts used during our analyses would possibly result in different outcomes, as our sensitivity analysis revealed. When using the more lenient definition of 2 days or less with pain as ‘recovered’, the picture was more positive, with only 13% of patients not recovering by this standard. The major difference in this sensitivity analysis was that the group “sustained recovery” increased from 20% to 48%, indicating that much of the experienced pain reported was 1 or 2 days of the week only.

Whichever definition is being used, one conclusion remains; LBP is a fluctuating condition. The commonly used descriptions of “acute” and “chronic” therefore seem obsolete, as the acute event is most likely part of a persistent condition.

Our analysis was simplistic, in that we did not take into account any background variables. Had we stratified the analysis on base-line variables, it is quite possible that we would have obtained specific course profiles with varying recovery profiles for specific background variables.

### Clinical implications

A previous report from this same study population showed how these patients could be divided into four clusters with distinctive courses and other specific characteristics [8]. These clusters were obtained in a process where each individual curve was described mathematically, thus the clusters were formed on the basis of similar development over time. Although this mathematical method seems to have opened the door for further fruitful sub-population studies, the results were not immediately clinically applicable.

In the present study, we have analyzed the same individuals’ data, but this time looking for a number of pre hoc defined patterns of recovery. In fact, this analysis was done visually, in such a way as it would be done by a clinician, if he/she were to collect this type of information on his own patients. The present study therefore adds a perspective on the issue of clinical course t more clinically relevant.

It is, of course, difficult in clinical practice to systematize clinical observations, and particularly difficult to do this in a longer perspective. There are several reasons for this. In clinic, during an ordinary working week, different types of conditions are treated at different points in their clinical courses. The treatment programs will differ between patients as will their compliance. Some patients will vanish before the treatment is considered completed, others will remain until discharged. Some will return with a new pain episode others will not. The method of elucidating outcome may not be systematic, objective or even relevant. Adding to this, information concerning the development of the condition is usually only available when the patient is undergoing care. Perhaps this is why the success stories stick best in memory. However, 20% of patients experienced a relapse after recovery and unless these patients contacted their chiropractor, this development would be unknown to the clinician. In fact, only about one fifth of all patients could be classified as having experienced early and sustained recovery, the outcome chiropractors and patients wish to see. Without tracking patients after treatment, a clinician is unable to get this information.

As our results show that patients with non-specific LBP will have a fairly mixed course pattern, it is recommended that, in research, more emphasis is placed on the importance of the clinical course rather than on the single outcome (“cure”). Past studies have shown unequivocally that past history of LBP is a strong determinant for future LBP [22, 23]. It is therefore important not to consider an event of LBP as “the” problem. In communicating with the patient, the fact that most LBP comes and goes should be emphasized, and that relapses are to be expected, regardless treatment. This is also in line with guideline recommendations for the management of non-specific LBP [24, 25].

Further, since only about one fifth of patients could be considered to experience a sustained favorable course, we think it important that this message be communicated among colleagues and chiropractic undergraduates, to avoid unrealistic hopes and professional disappointment. Further, it would be relevant to attempt to identify the features of the subgroups identified in this study, in order to learn more about if and how they should be treated.

## Conclusions

The’ typical’ chiropractic patient, in the sense that there should be a large (majority) group with a similar course, did not exist. Only a fifth of patients with LBP treated by chiropractors and then followed weekly for 6 months recovered and sustained recovery. Knowledge of the real recovery patterns should lead to realistic expectations regarding the course of LBP, which would probably benefit both chiropractors and patients. Clinicians may use these findings to inform patients that improvement after treatment is expected, but sustained recovery is not a common feature of LBP.

From a research perspective, it is important to take into account the clinical course when studying effect of treatment and clinical outcome, as it differs from the end-of study measurements.
